# Pediatric laparoscopy: Facts and factitious claims

**DOI:** 10.4103/0971-9261.72434

**Published:** 2010

**Authors:** V. Raveenthiran

**Affiliations:** Division of Pediatric Surgery, Rajah Muthiah Medical College, Annamalai University, Chidambaram, Tamil Nadu, India

**Keywords:** Hospital stay, postoperative pain, pediatric laparoscopy, wound cosmesis, laparotomy, abdominal surgery, minimal invasive surgery, children, infants

## Abstract

**Background:**

Pediatric laparoscopy (LS) is claimed to be superior to open surgery (OS). This review questions the scientific veracity of this assertion by systematic analysis of published evidences comparing LS versus OS in infants and children.

**Materials and Methods:**

Search of PubMed data base and the available literature on pediatric LS is analyzed.

**Results:**

One hundred and eight articles out of a total of 426 papers were studied in detail.

**Conclusions:**

High quality evidences indicate that LS is, at the best, as invasive as OS; and is at the worst, more invasive than conventional surgery. There are no high quality evidences to suggest that LS is minimally invasive, economically profitable and is associated with fewer complications than OS. Evidences are equally distributed for and against the benefits of LS regarding postoperative pain. Proof of cosmetic superiority of LS or otherwise is not available. The author concludes that pediatric laparoscopy, at the best, is simply comparable to laparotomy and its superiority over the latter could not be sustained on the basis of available scientific evidences. Benefits of laparoscopy appear to recede with younger age. Concerns are raised on the quick adoption, undue promotion and frequent misuse of laparoscopy in children.

“There are a few members of our profession who exploit fashions and play up to the public demand for them because it pays so to do.”

Sir Robert Hutchison (1925)

## INTRODUCTION

“Peeping Toms” are condemned, but “peeping surgeons” are hailed. The entire surgical fraternity now appears to be under the spell of “Laparoscopy.” Although laparoscopes are known for more than 100 years, only during the last two decades an unprecedented hype is perceptible among the public as well as among professionals.[1] Newer technologies are always welcome as they are likely to benefit humanity. Nevertheless, as Sir Robert Hutchison remarked, “It is always well, before handing the cup of knowledge to the young, to wait until the froth has settled.”[2] Any new technology should be critically examined, practically explored and adequately experienced before incorporating it into routine practice. Have we ever approached pediatric laparoscopy analytically? Have we waited enough for the froth to settle? Pediatric laparoscopy appears to be too quickly adopted, unduly advertised and frequently misused by individual surgeons for their personal profit.

Enthusiasts of pediatric laparoscopy claim that it is minimally invasive, less painful, cost-effective, cosmetically more appealing and is associated with shorter hospital stay and fewer complications. In this communication, I intend to analyze whether these claims are sustainable by the available research evidences.

## METHODS AND MATERIALS

The PubMed database was electronically searched for English language literature on pediatric laparoscopy using the search string {laparoscopy OR “minimally invasive”}. Search was restricted to the last 20 years (1990-2010 August) as it corresponds to the time of popularization of the technique. Search and analysis were limited to the pediatric age group. For the purpose of this review, ‘pediatric’ is defined as ‘from newborn to 12 years of age’. Only laparoscopic procedures were considered for analysis; thoracoscopy and other endoscopic procedures were excluded.

Papers were categorized according to their “quality of evidence” (QoE). Although the classification is based on the principles of the Oxford Center for Evidence-Based Medicine (CEBM),[3] it is not the same as the “Level of Evidence” proposed by the center. The complex system of CEBM has been simplified in Table 1. In the presence of higher quality evidence, papers with lower QoE become scientifically invalid and, hence, they were ignored. When there were more than two papers of the same QoE but with conflicting conclusions, each of them was carefully dissected further for methodological flaws and statistical power.[4]

**Table 1:** Hierarchy of the Quality of Evidence (QoE)*

Quality of Evidence†	Definition
Class 1	Systematic Reviews / Meta-analysis of RCTs
Class 2	Single- or Double-Blinded RCT (or)
	Prospective Multicentric study
Class 3	RCT without blinding
Class 4	Prospective Controlled study without randomization
	(or) Cohort studies
Class 5	Quasi-experimental studies (or)
	Retrospective case-control studies
Class 6	Descriptive case series
Class 7	Isolated Case reports
Class 8	Expert Opinion / Anecdotes / Consensus of a group

RCT- Randomized Controlled Trials,

* “Quality of Evidence (QoE)” is not the same as “Level of Evidence” proposed by Oxford Center for Evidence Based Medicine (CEBM). But both are analogous.

† Class 1 is of highest quality evidence and class 8 is the lowest quality. In the text, “Quality of Evidence Class 1” is abbreviated as QoE-C1 and so forth.

## RESULTS

**Figure 1:**
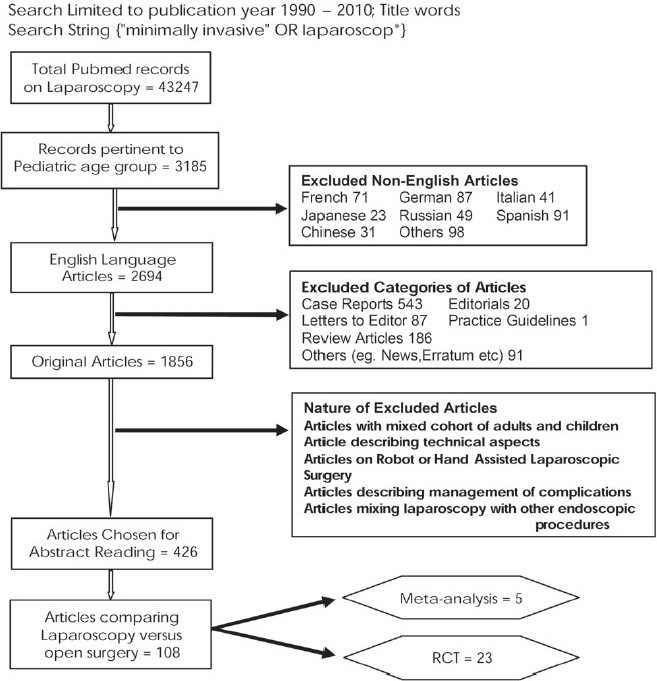
Quorum analysis of the PubMed literature on pediatric laparoscopy

Figure 1 summarizes the PubMed search results. Abstracts of 426 articles were read and appropriate articles were chosen. There were 108 articles that compared open versus laparoscopic procedures in children. Among these, there were five meta-analysis and 23 randomized controlled trials (RCTs). Full texts of the chosen articles were read critically.

## DISCUSSION

### Is Pediatric Laparoscopy Minimally Invasive?

There cannot be anything more folly than to label laparoscopy as “minimally invasive.” Invasiveness of a surgical procedure is determined not by the length of the skin incision but by the stress response elicited in the host. “Surgical stress” is a convenient term referring to the sum total of all physiological changes that causes disequilibrium and threatens homeostasis of an individual following a surgical operation.[5] The adjective “surgical” is misleading because stress caused by anesthesia is often inseparable from that of surgery. It is true that longer incisions, by virtue of greater tissue trauma, produce more stress than smaller incisions (QoE-C4).[6] However, isolated analysis of individual stressors is fallacious because stress is the sum total of the effect of all possible stressors. Therefore, in addition to the length of the skin incision, other stressors such as the type of anesthesia, duration of surgery, amount of surgical dissection, degree of tissue desiccation, adequacy of pain control etc. should be considered *en bloc* while analyzing the stress of laparoscopic surgery (LS). Notwithstanding the difference in the mode of accessing viscera, the actual intraabdominal dissections of LS cannot be any different from that of open surgery (OS). During the period of the learning curve, the operative time is significantly longer for LS than for OS. Theoretically, even after the learning curve, the operative time cannot be significantly shorter for LS because the actual surgical dissections are similar to that of OS. Two meta-analyses of pyloromyotomy[7,8] and one of appendectomy[9] concluded the operative time to be similar in OS and LS (QoE-C1). Nonetheless, RCT of pyeloplasty (QoE-C3)[10] and inguinal hernia repair (QoE-C2)[11,12] suggested LS to be lengthier than OS. Laparoscopy necessitates general endotracheal inhalation anesthesia even for those procedures, such as herniotomy, which can otherwise be performed under regional nerve block. It is well known that general inhalation anesthesia is more stressful than regional blocks with sedation.[13] Thus, LS is not less invasive than OS with regard to anesthesia-related stress, length of operation and magnitude of tissue dissection.

**Figure 2:**
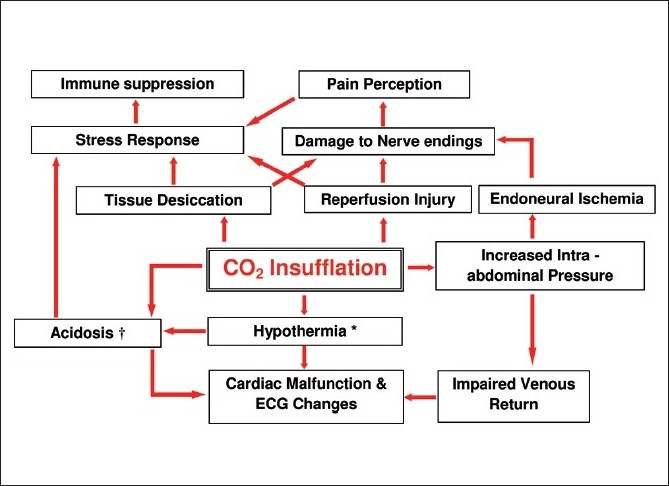
Physiological changes induced by CO_2_ pneumoperitoneum during laparoscopic surgery in children. *Hypothermia also contributes to stress response. ^†^Acidosis also contributes to postoperative pain

Stressors are not only physical but also chemical and emotional. Chemical insult caused by carbon dioxide (CO_2_) is unique for laparoscopy. The five cardinal perils of CO_2_ pneumoperitoneum are abdominal compartment syndrome,[14] hypercarbia,[15] respiratory acidosis,[16] tissue desiccation and hypothermia.[17,18] They together contribute significantly to several physiological changes [Figure 2]. For example, electrocardiograms (ECG) of children undergoing laparoscopic appendectomy showed significant QT dispersion and P-wave depression during CO_2_ insufflation (QoE-C4).[19] Although these ECG changes were reversible, it is a cause of concern that they predict severe atrial or ventricular arrhythmias. High intraabdominal pressure of the pneumoperitoneum causes splanchnic ischemia during laparoscopy and reperfusion occurs during deflation of gas. This ischemic-reperfusion sequence was recently shown to generate reactive oxygen species, thereby increasing the oxidative stress and the consumption of plasma antioxidants in children undergoing LS (QoE-C4).[20]

Serum levels of certain chemicals are elevated in response to surgical stress. The degree of the overall stress can be deduced by a quantitative estimation of these chemical markers. Elevated blood glucose level is a reliable indicator of stress. Dave *et al*.[21] studied the blood glucose changes in 120 children randomized to LS and OS. With infusion of a non-dextrose-containing intravenous fluid (ringer lactate), blood glucose was significantly higher in the LS group. However, this difference was masked when a dextrose-containing fluid was used. Conclusions of this RCT indicate that LS elicits more stress response than OS (QoE-C3). McHoney *et al*.[22] randomized 40 children undergoing fundoplication and studied the plasma levels of inflammatory markers such as malondialdehyde, nitrates, nitrite, interleukin (IL)-10, IL-6 and tumor necrosis factor alfa (TNF-a). They also studied monocyte class II major histocompatibility complex expression and IL-1 receptor antagonist (IL-1ra, an anti-inflammatory cytokine). The trial concluded that the circulating markers of inflammation and secondary oxidative stress were not significantly different between the LS and the OS groups (QoE-C3). Bozkurt *et al*.[23] estimated the serum levels of prolactin, cortisol, IL-6, glucose, insulin, lactic acid and epinephrine among children with acute abdominal pain. They concluded that surgical stress and trauma imposed by laparoscopy were similar to that of laparotomy (QoE-C3). Simon *et al*.[24] found that inflammatory markers such as body temperature, leukocyte count, hematocrit and serum levels of C-reactive protein (CRP), IL-6, TNF, sTNF-R, IL-1ra, sIL-2r and IL-8 were no different in children undergoing laparoscopic or open appendectomy (QoE-C3). An assessment of surgical stress using CRP and leukocyte count did not reveal any difference between children undergoing laparoscopy-assisted and posterior sagittal anorectoplasty for imperforate anus (QoE-C4).[25] Similarly, IL-6 and CRP levels did not differ significantly among children undergoing laparoscopy versus open appendectomy (QoE-C4).[26] Solitary studies on pull-through for Hirschsprung disease,[27] pyloromyotomy[28] and neonatal surgery[29] claimed a significantly lesser amount of stress markers in LS than in OS. However, all the three are retrospective reviews and hence can be ignored because of the inferior quality evidence (QoE-C5). It is now evident that LS is, at the best, as invasive as OS and is, at the worst, more invasive than conventional surgery.

### Is Laparoscopy Cosmetically Superior?

Smaller scars are obviously more pleasing. Irrefutability of this contention, perhaps, precluded designing of proper scientific studies to address the cosmetic superiority of LS. Small scars of LS persuade many authors to end their papers proclaiming “excellent cosmetic results,” even when there are no data to support it. Blinded RCTs of inguinal herniotomy[11] and pyloromyotomy[30] concluded that scar cosmesis was similar between the LS and the OS groups (QoE-C2). A questionnaire survey on the cosmetic outcome of pyloromyotomy using a “willingness-to-pay” model was done. As 85% of the parents were willing to pay more money to have the scar of laparoscopic pyloromyotomy, the authors concluded that it is superior to the scar of open pyloromyotomy (QoE-C4). Findings of this otherwise well-conducted study are marred by inappropriate control group, poor quality of evidence and a possible selection bias in the photographic templates used. The willingness-to-pay model will be fallacious when applied to healthy volunteers instead of actual patients - as it is in this study. Further, transumbilical pyloromyotomy is well known to produce better scar than laparoscopy (QoE-C5).[31–33]

In the absence of robust scientific data, which is unlikely to evolve, several concerns need to be addressed philosophically. First of all, scar is not the end point of any abdominal operation. Too much emphasis on scar cosmesis can be nothing but scientific blasphemy. Scars are important only if they can be easily seen in the public or if they cause restriction of physical movements. Abdominal scars, more so the inguinal ones,[34] can be effectively and elegantly concealed, even while swimming seminude, by appropriate attire. Therefore, what is the big deal of a small abdominal scar, unless clients are brainwashed by inappropriate propaganda? How many of those who were willing to pay an additional out-of-pocket amount for a laparoscopic pyloromyotomy scar[33] would do so if they had been unbiasedly counseled as to the practical implications of a 3-cm scar in the abdomen? Secondly, good principles go a long way. Even the scar of a lengthy wound can be made invisible by adhering to sound surgical principles. If a plastic surgeon can inflict an acceptable scar in the face, cannot a pediatric surgeon replicate it in the abdomen? Finally, the apprehension of an “ugly laparotomy scar” is a naive transcription of adult surgical concept into pediatric practice. At least for the last three decades, we know that wounds heal differently in children and the process is age dependent.[35,36] The younger the child, the more invisible will be the scar. In its extreme form, fetal wounds heal absolutely scarlessly.[37] Promoting laparoscopy in neonates and infants, drawing analogies from the adult literature on scar cosmesis, can be due to anything but insightfulness.

### Is Laparoscopy Painless?

Many laparoscopists subconsciously use the term “painless” to mean “less painful”. Obviously, the smaller the skin incision, the lesser is the expected pain. But, in reality, for two reasons, laparoscopy defies this simple logic. Firstly, nociceptors are not only stimulated by mechanical trauma of the scalpel but also by noxious chemicals, high pressure and extremes of temperature. The abdominal cavity is stretched by the pressure of gas insufflation, leading to endoneural ischemia of the phrenic and peritoneal nerves.[38] Additionally, acidic intraperitoneal milieu caused by the dissolution of CO_2_ and dryness caused by gas inflow irritates the nerve endings. Consequently, CO_2_ pneumoperitoneum appears to cause more pain than what is due to the parietal incision. Secondly, pain is a perceived subjective phenomenon that is difficult to quantify. It is easily modified by even simple preoperative suggestions such as “small incision.”

Finnish surgeons[11] have reported the median pain score on the second postoperative morning to be significantly higher after laparoscopic hernia repair than after open herniotomy (QoE-C2). Lejus *et al*.[39] noted referred shoulder pain in 35% of the children undergoing laparoscopic appendectomy, while only 10% of those who underwent open surgery experienced it (QoE-C2). Two more RCTs - one on appendectomy[40] and another on pyeloplasty[10] - did not find any significant difference in pain scores and analgesic requirements between the LS and the OS groups (QoE-C3). Although one double-blinded RCT[30] noted slightly higher pain scores (less pain) in laparoscopic pyloromyotomy, there was no difference between the groups in the analgesic requirement (QoE-C2). Moreover, the authors themselves acknowledge a possible inter- and intra-observer variability in accessing pain of neonates and infants who cannot express it.

A single-blinded RCT on appendectomy[41] and inguinal herniotomy[12] showed significantly lesser pain and lesser analgesic requirement in LS than in OS (QoE-C2). Two further RCTs - one on pyloromyotomy[42] and another on varicocelectomy[43] - reached a similar conclusion (QoE-C3). As evidences are inconclusive, a formal meta-analysis or a well-designed double-blind RCT with higher statistical power is required to settle the issue. Nevertheless, we can safely conclude that LS, contrary to the claims of laparoscopists, causes considerable pain, although less than that of OS.

### Is Laparoscopy Economical?

Despite the absence of reliable financial analysis by economic experts, enthusiasts of laparoscopy frequently claim that it is cost-effective. Early discharge from hospital is frequently cited as proof of this assertion. None of the available studies take into consideration all the components of financial analysis. Laparoscopic appendectomy costs more in terms of OR time and management of complications.[44] The total cost of LS was still higher than OS, despite a marginal offset by shorter hospital stay in the former (QoE-C3). In another RCT of pyeloplasty,[10] LS was slightly more expensive than OS, although this difference was not statistically significant (QoE-C3). Several retrospective studies of appendectomy,[45–47] splenectomy,[48] nephrectomy[49] and herniotomy[50] acknowledged a higher cost of LS as compared with OS (QoE-C5).

A retrospective study of fundoplication[51] found that laparoscopy costs more in terms of anesthesia, sterilization and prolonged operating time. As this was offset by the cost of equipment, prolonged hospital stay and pharmacy bill in the OS group, the two groups did not differ significantly in the total cost (QoE-C5). A similar conclusion (QoE-C5) was reached by studies on adrenalectomy[52] and pyloromyotomy.[53] Cervellione *et al*.[54] claimed that laparoscopic nephrectomy was 54% less expensive than OS. This study suffers from inferior quality of evidence (QoE-C5). It is also interesting to note that the mean cost of disposable instruments was 13-times higher in LS than in OS, but the cost of hospital stay was only three-times higher in OS than in LS. We can conclude that there are no high-quality evidences to claim the cost-benefits of LS.

### Is Laparoscopy Associated with Fewer Complications?

Cosmesis and cost are not the end points of any abdominal surgery. Efficacy of correcting pathology and restoration of physiological function are of paramount importance. LS can be accepted only when it is conclusively shown to be more effective, with fewer complications, than OS. Two independent meta-analyses[7,8] have conclusively shown that incomplete pyloromyotomy was more common in LS than in OS (QoE-C1). Alarmingly, the rate of redo pyeloplasty was doubled in the LS group as compared with the OS group (QoE-C1).[55] Although wound infection and ileus were lower with laparoscopic appendectomy, intraabdominal abscesses were more frequent in this group (QoE-C1).[9] In the same study, a subgroup analysis did not show any difference in the four common complications of appendectomy between the LS and the OS groups. Manometry of children undergoing anorectoplasty for imperforate anus suggested a slight improvement in the resting pressure of anal canal in the LS group than in the OS group.[56] However, the same study could not show any difference in the clinical scoring between the two groups (QoE-C3). In a large, nationwide, multicentric study[57] involving 89,497 pediatric appendectomies, there was no difference in length of stay and 28-day readmission rates and the 30- and 365-day mortality were similar in the LS and OS groups (QoE-C5). Among children undergoing the Kasai procedure for biliary atresia,[58] the rate of early failure was 66.6% in the LS versus 38.5% in the OS (QoE-C5) group. The only exception to the gloomy picture of laparoscopy is the report of Podkamenev *et al*.,[43] who noted that wound complication and scrotal edema were lower with laparoscopic varicocelectomy than with OS (QoE-C3). Overwhelming evidences indicate that LS, in a majority of procedures, has similar complication rates as that of OS. Higher complications of LS in certain operations such as pyloromyotomy are certainly a source of concern.

### On the Origin of Fashions and Faddism

It is surprising to learn as to how laparoscopists have sustained their claims despite the lack of high-quality evidence. This faddism is reflected in distorted interpretation of study results in some of the published papers. For example, a double-blind multicentric study of pyloromyotomy[59] concluded in favor of laparoscopy because of shorter hospital stay. The mean duration of stay was 43.8 h for LS versus 33.6 h for OS, and this difference was statistically significant at a P-value of 0.027. In their enthusiasm to support laparoscopy, the authors have ignored the fact that statistical significance is not the same as clinical significance. In the given example, had the authors used “days” instead of “hours,” the two groups would not have differed significantly. Measuring hospital stay in “days” is more practical than doing so in “hours.” To understand the source of such faddism, we need to have insight into the influence of industry, insurers and individuals. Although it is possible to perceive, proof of this is difficult. In the initial phases of a new technology, individual surgeons promote it with an ulterior motive of distinguishing themselves from their professional competitors. At a later phase, “once a treatment has become fashionable, the public demand for it helps to keep it alive”.[2]

William Ladd founded pediatric surgery with a notion that children are not miniature adults. It is disheartening to see this principle being flouted by enthusiasts of laparoscopy. Quick discharge from the hospital may be important in adults, who may incur loss of wages unless they return to work early. Is it not inanity to apply this concept to a toddler? What is he going to do by returning home early other than play around? Emerging scientific evidence indicates that benefits of laparoscopy recede with younger age (QoE-C5).[60] Even CO_2_ elimination appears to be age dependent, with younger children absorbing proportionately more CO_2_ than older individuals.[61] But, who is paying attention to these concerns and analyzing the evidences carefully? Every one enjoys jumping on the bandwagon. Faddists have made laparoscopy fashionable. As Sir Robert Hutchison remarked, “One should not envy the fashionable doctor; rather should one wonder at him.”[2]
